# Prognostic parameters on baseline and interim [^18^F]FDG-PET/computed tomography in diffuse large B-cell lymphoma patients

**DOI:** 10.1097/MNM.0000000000001664

**Published:** 2023-01-30

**Authors:** Sándor Czibor, Robert Carr, Francisca Redondo, Chirayu U. Auewarakul, Juliano J. Cerci, Diana Paez, Stefano Fanti, Tamás Györke

**Affiliations:** a Department of Nuclear Medicine, Medical Imaging Centre, Semmelweis University, Budapest, Hungary; b Department of Hematology, Guy’s and St. Thomas’ Hospital, King’s College London, London, UK; c Oncologic Clinic, Fundación Arturo Lopez Perez, Santiago, Chile; d Faculty of Medicine and Public Health, HRH Princess Chulabhorn College of Medical Science, Chulabhorn Royal Academy, Bangkok, Thailand; e PET/CT Department at Quanta Diagnóstico e Terapia, Curitiba, Brazil; f Nuclear Medicine and Diagnostic Imaging Section, Division of Human Health, Department of Nuclear Sciences and Application, International Atomic Energy Agency, Vienna, Austria; g Metropolitan Nuclear Medicine, Policlinico S. Orsola, University of Bologna, Bologna, Italy

**Keywords:** diffuse large B-cell lymphoma, Deauville score, PET, metabolic tumor volume, ratio PET

## Abstract

2-[^18^F]fluoro-2-deoxy-d-glucose PET/computed tomography ([^18^F]FDG-PET/CT) is a widely used imaging method in the management of diffuse large B-cell lymphomas (DLBCL). Our aim was to investigate the prognostic performance of different PET biomarkers in a multicenter setting.

**Methods:**

We investigated baseline volumetric values [metabolic tumor volume (MTV) and total lesion glycolysis (TLG), also normalized for body weight] segmented with three different methods [>SUV4 (glob4); 41% isocontour (41pc), and a gradient-based lesion growing algorithm (grad)] and interim parameters [Deauville score, maximal standardized uptake value (ΔSUVmax), modified qPET, and ratio PET (rPET)] alongside clinical parameters (stage, revised International Prognostic Index), using 24-month progression-free survival as the clinical endpoint. Receiver operating characteristics analyses were performed to define optimal cutoff points for the continuous PET parameters.

**Results:**

A total of 107 diffuse large B-cell lymphoma patients were included (54 women; mean age: 53.7 years). MTV and TLG calculations showed good correlation among glob4, 41pc, and grad methods; however, optimal cutoff points were markedly different.

Significantly different PFS was observed between low- and high-risk groups according to baseline MTV, body weight-adjusted (bwa) MTV, TLG, bwaTLG, as well as interim parameters Deauville score, ΔSUVmax, mqPET, and rPET. Univariate Cox regression analyses showed hazard ratios (HRs) lowest for bwaMTVglob4 (HR = 2.3) and highest for rPET (HR = 9.09). In a multivariate Cox-regression model, rPET was shown to be an independent predictor of PFS (*P* = 0.041; HR = 9.15). Combined analysis showed that ΔSUVmax positive patients with high MTV formed a group with distinctly poor PFS (35.3%).

**Conclusion:**

Baseline MTV and TLG values and optimal cutoff points achieved with different segmentation methods varied markedly and showed a limited prognostic impact. Interim PET/CT parameters provided more accurate prognostic information with semiquantitative ‘Deauville-like’ parameters performing best in the present study.

## Introduction

Diffuse large B-cell lymphoma (DLBCL) is a clinically, pathologically, and molecularly heterogeneous hematological malignancy, considered the most common subtype of non-Hodgkin lymphomas [1]. In its initial clinical staging, the utility of 2-[^18^F]fluoro-2-deoxy-d-glucose (FDG) PET/computed tomography (PET/CT) examination has gained vast evidence and is incorporated in current recommendations [2].

Aside from well-researched clinical, pathological, and molecular prognostic factors, several FDG-PET/CT-based biomarkers have emerged in the last decade, also carrying prognostic information (beyond its inherent prognostic value in defining the clinical stage of DLBCL).

Of these parameters, metabolic tumor volume (MTV) and total lesion glycolysis (TLG) have shown promise to yield added prognostic value to established clinical scores, for example, the International Prognostic Index (IPI) and its modifications, the revised IPI (R-IPI) and the National Comprehensive Cancer Network-IPI (NCCN-IPI) [3–8].

Beyond its utility as baseline investigation, FDG-PET/CT plays an important role in the evaluation of treatment response at the end of therapy, or even in an early assessment, interim setting. Robust and widespread evaluation criteria based on the Deauville-five-point scale have been established to decide the presence or absence of complete metabolic remission [9,10]. Aside from the ordinal Deauville score, continuous values have been investigated in high-grade lymphomas, most notably the proportional decrease of lesion maximal standardized uptake value (ΔSUVmax) and, to a lesser extent, semiquantitative ‘Deauville-like’ parameters, such as qPET and ratio PET (rPET) [11–18].

Our aim was to investigate the prognostic performance of baseline volumetric values (MTV and TLG) and interim parameters (Deauville score and semiquantitative) derived from the FDG-PET/CT scans of DLBCL patients in a multicenter setting.

## Methods

We investigated the baseline and interim PET/CT scans of DLBCL patients included in a prospective, multicentric study coordinated by the International Atomic Energy Agency (IAEA) who received R-CHOP (rituximab combined with cyclophosphamide, doxorubicin, vincristine, and prednisolone) immunochemotherapy. The study design was elaborately described before [19], this time a reduced number of patients was included in our sample after the following exclusion criteria: treatment other than R-CHOP; studies performed on a stand-alone PET scanner; studies performed on different PET/CT scanners in baseline and interim setting; missing or compromised imaging data; event-free follow-up lasting less than 24 months. Ten centers in the same number of countries (Brazil, Chile, Hungary, India, Italy, Pakistan, the Philippines, South Korea, Thailand, and Turkey) participated in the IAEA study. The research was approved by the respective ethical review board of each participating center and all subjects signed an informed consent form.

Clinical stage was determined by the baseline PET/CT scans according to the Lugano criteria and R-IPI was calculated for each patient [2,7]. The volumetric and semiquantitative evaluation of the PET/CT images was performed by central review. Lymphoma lesions on baseline PET images were delineated with three different methods: (1) >SUV4 (glob4); 41% isocontour VOI around the local maximum point (41pc); a vendor-specific gradient-based lesion growing algorithm (grad), performed with Mediso InterView Fusion software (Mediso Medical Imaging Systems, Budapest, Hungary). MTV was calculated as the sum of all lymphoma lesions’ volume on PET images, and TLG was determined as the sum of the product of each lesion’s metabolic volume and SUVmean. Both MTV and TLG values were normalized for patient body weight, thus introducing body weight-adjusted (bwa) MTV and bwaTLG values. Receiver operating characteristics (ROC) analyses were performed to define optimal cutoff points for MTV, TLG, bwaMTV, and bwaTLG for the three different segmentation methods.

Interim PET/CT scans were analyzed visually according to the Deauville criteria, resulting in Deauville scores 1–5, and semiquantitatively. Deauville score 5 was defined as lesion SUVmax three times over liver SUVmax. The semiquantitative evaluation methods included the proportional change in SUVmax in percents between the baseline and interim scans (ΔSUVmax) and two semiquantitative ‘Deauville-like’ parameters for which a 3 cm diameter spheric VOI was placed in the unaffected part of the right liver lobe. Modified qPET (mqPET) is the proportion of the hottest lesion’s SUVpeak (the SUVmean of the hottest 1 cm^3^ in the lesion VOI) and the SUVmean of the liver VOI – the original qPET value, described first by Hasenclever *et al*. in pediatric Hodgkin’s lymphoma used the mean SUV of the hottest four adjacent voxels in the lesion [13]. Our use of the 1-cm^3^ SUVpeak was based on the lack of adequate software as well as the hypothesis that in adult patients this volume would not lead to considerable distortion in the results. The rPET, as described before, is the proportion of the SUVmax in the hottest lesion and the SUVmax in the liver reference VOI [16,17].

When establishing the diagnostic performance of the above different prognostic biomarkers, 24-month progression-free survival was the clinical endpoint. Statistical calculations were performed in the R environment (The R Foundation, https://www.r-project.org) with R Studio software (RStudio PBC; Boston, Massachusetts, USA).

## Results

### Patient characteristics

A total of 107 patients were included in the present study (mean age: 53.7; range: 16–83 years) with 53 women and 54 men among them. The majority of patients were from Hungary (57) and Chile (36), while 8, 4, and 2 of them were from Thailand, the Philippines, and Italy, respectively. 58% of the patients presented with advanced-stage disease. Further patient information is provided in Table 1.

**Table 1:** Patient characteristics and clinical data

Characteristic	*n* = 107 (100%)
Sex	
Male	54
Female	53
Age	
Range	16–83
Median	56
>60 years	44 (41%)
Performance status	
0–1	83 (78%)
2–4	24 (22%)
Stage	
I	16 (15%)
II	29 (27%)
III	19 (18%)
IV	43 (40%)
R-IPI	
Good	24 (22%)
Intermediate	48 (45%)
Poor	35 (33%)
Timing of interim PET/CT	
After two cycles of R-CHOP	90 (84%)
After three cycles of R-CHOP	17 (16%)

CT, computed tomography; R-IPI, Revised International Prognostic Index; R-CHOP, rituximab combined with cyclophosphamide, doxorubicin, vincristine, and prednisolone.

### Comparison of volumetric parameters achieved by different delineation methods

**Fig. 1:**
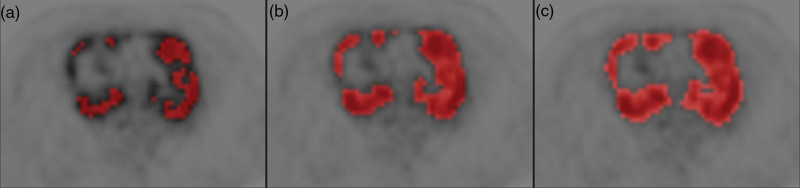
Transaxial PET images with fused mediastinal lymphoma MTV VOIs. Different MTV segmentation techniques yielding different MTVs: (a) glob4 with 294 cm^3^, (b) 41pc with 579 cm^3^, and (c) grad with 798 cm^3^. bwaMTV, body weight-adjusted metabolic tumor volume; MTV, metabolic tumor volume; VOI, volume of interest.

MTV and TLG calculations showed a good correlation among glob4, 41pc, and grad methods (Table 2), despite occasionally resulting in markedly different volumes (Fig. 1). ROC analyses yielded markedly different optimal cutoff points for MTV, TLG, bwaMTV, and bwaTLG with the three different segmentation methods (Table 3). Areas under the curve (AUCs) did not show a significant difference between MTV vs. bwaMTV and TLG vs. bwaTLG with the corresponding segmentation methods, the values ranging between 0.62 and 0.68 (Table 3). More diverse values in sensitivity, specificity, positive and negative predictive values, and diagnostic accuracy could be observed, primarily among the same volumetric parameters with different segmentation methods and not between traditional and bwaMTV or TLG.

**Table 2:** Pearson-correlation coefficients between volumetric parameters by different segmentation methods

MTV	41pc	grad	TLG	41pc	grad
glob4	0.872	0.849	glob4	0.981	0.984
41pc		0.962	41pc		0.993

bwa, body weight-adjusted; glob4, >SUV4 method; grad, method using a gradient-based lesion growing algorithm; MTV, metabolic tumour volume; TLG, total lesion glycolysis; 41pc, 41% isocontour VOI method.

**Table 3:** Cutoff values, areas under the curve, and diagnostic performance of volumetric parameters by different segmentation methods

	MTV			TLG			bwaMTV			bwaTLG		
	glob4	41pc	grad	glob4	41pc	grad	glob4	41pc	grad	glob4	41pc	grad
Cutoff	122.5	257.5	334.9	714.7	1207	2112	2.55	2.68	7.84	15.5	13.2	53.3
AUC	0.63	0.65	0.66	0.62	0.64	0.65	0.65	0.67	0.68	0.63	0.65	0.65
Sensitivity (%)	77.8	74.1	63	81.4	77.8	62.9	66.7	77.8	55.6	70.4	77.8	51.9
Specificity (%)	47.5	56.3	70	41.3	51.3	62.5	56.3	57	76.2	51.3	48.8	73.8
PPV (%)	33.3	36.4	41.4	31.9	35	36.2	34	36.2	44.1	32.8	33.9	40
NPV (%)	86.3	86.5	84.8	86.8	87.2	83.3	83.3	89.1	83.6	83.7	86.7	81.9
Accuracy (%)	55.1	60.7	68.2	51.4	57.9	62.6	58.9	61.9	71	56.1	56.1	68.2

AUC, area under the curve; bwa, body weight-adjusted; MTV, metabolic tumor volume; TLG, total lesion glycolysis; glob4, >SUV4 method; 41pc, 41% isocontour VOI method; grad, method using a gradient-based lesion growing algorithm; PPV, positive predictive value; NPV, negative predictive value.

### Prognostic value of baseline and interim biomarkers

With the aim of a more transparent data presentation, only the >SUV4-method-based (glob4) volumetric values (MTV, TLG, bwaMTV, and bwaTLG) are presented, as it is considered the most easily reproducible segmentation method.

ROC analyses were performed to define optimal cutoff points for interim PET semiquantitative values, yielding values of −77.22%, 1.32, and 1.54 for ΔSUVmax, mqPET, and rPET, respectively. AUCs, sensitivity, specificity, positive and negative predictive values, and diagnostic accuracy of interim parameters are detailed in Table 4.

**Table 4:** Cutoff values and diagnostic performance of interim parameters

	Deauville scores 1–3/4–5	ΔSUVmax	mqPET	rPET
Cutoff		−71.22%	1.32	1.54
AUC		0.66	0.73	0.71
Sensitivity (%)	59.3	48.1	59.2	55.6
Specificity (%)	83.8	85	87.5	92.5
PPV (%)	55.2	52	61.5	71.4
NPV (%)	85.9	82.9	86.4	86
Accuracy (%)	77.6	75.7	80.4	83.2

AUC, area under the curve; mqPET, modified qPET; NPV, negative predictive value; PPV, positive predictive value; rPET, ratio PET; SUVmax, maximum standardized uptake value.

**Fig. 2:**
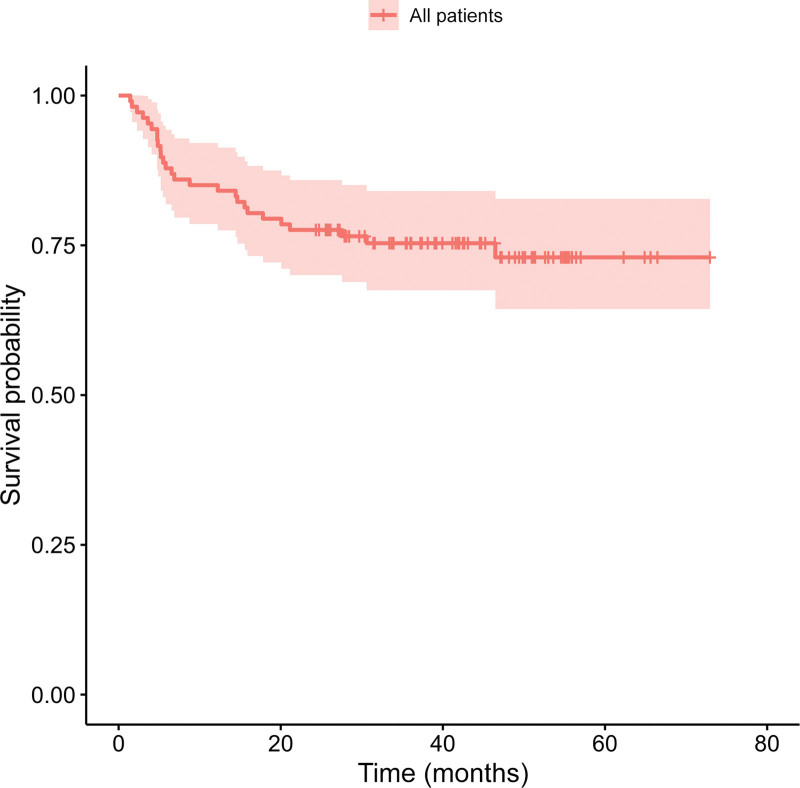
Progression-free survival curve of the patient population.

**Fig. 3:**
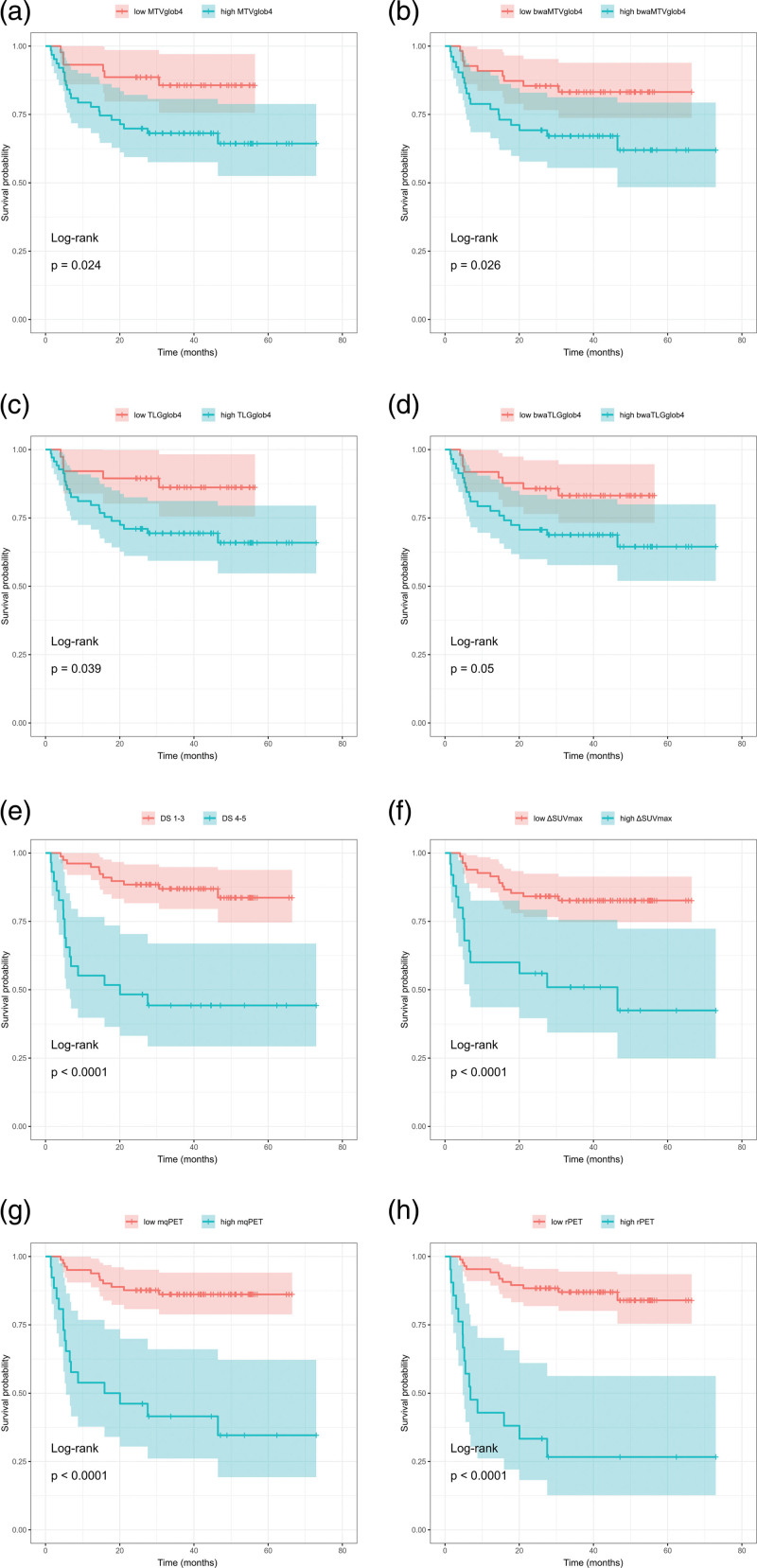
Kaplan–Meier curves of progression-free survival between low- and high-risk patient groups divided according to baseline MTV (a), bwaMTV (b), TLG (c), bwaTLG (d), and interim parameters Deauville score (e), ΔSUVmax (f), mqPET (g), and rPET (h). bwa, body weight-adjusted; MTV, metabolic tumor volume; mqPET, modified qPET; rPET, ratio PET; SUVmax, maximum standardized uptake value; TLG, total lesion glycolysis.

Progression-free survival in the whole cohort was 75% (Fig. 2). Interestingly, log-rank survival analysis did not show a significant difference between the PFS of early and advanced-stage patients (82% vs. 69%). Dividing the patients into two groups according to calculated optimal cutoffs or predefined values (in the case of Deauville score) resulted in significantly different PFS for baseline MTV, bwaMTV, TLG, bwaTLG, as well as interim parameters Deauville score (1–3 vs. 4–5), ΔSUVmax, mqPET, and rPET (Table 5 and Fig. 3).

**Table 5:** Twnety-four-month progression-free survival rates of low- and high-risk groups according to different parameters

	MTV (%)	bwaMTV (%)	TLG (%)	bwaTLG (%)	Deauville score (%)	ΔSUVmax (%)	mqPET (%)	rPET (%)
Low risk	86	83	87	84	86	83	86	86
High risk	67	66	69	67	45	48	38	29

bwa, body weight-adjusted; glob4, >SUV4 method; grad, method using a gradiant-based lesion growing algorithm; MTV, metabolic tumour volume; mqPET, modified qPET; SUVmax, maximum standardized uptake value; TLG, total lesion glycolysis; 41pc, 41% isocontour VOI method.

**Fig. 4:**
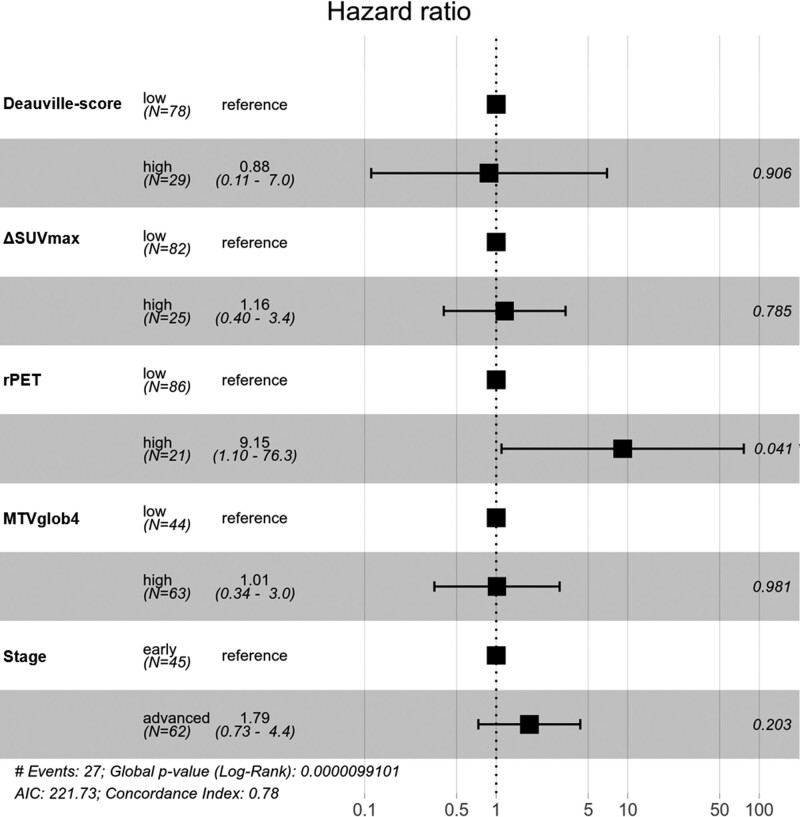
Multivariate Cox-regression model of progression-free survival including Deauville score, ΔSUVmax, rPET, MTV, and clinical stage. MTV, metabolic tumor volume; rPET, ratio PET; SUVmax, maximum standardized uptake value.

Univariate Cox-regression analyses showed a significant difference between low- and high-risk groups except for early/advanced stage and low/high bwaTLGglob4, with calculated hazard ratios (HRs) the lowest for bwaMTVglob4 (HR = 2.3) and the highest for rPET (HR = 9.09) among the remaining prognostic parameters (Table 6). In a multivariate Cox-regression model including Deauville score (1–3 vs. 4–5), ΔSUVmax, rPET, MTV, and clinical stage (early vs. advanced) only rPET was shown to be a significant independent predictor of PFS (*P* = 0.041; HR = 9.15) (Fig. 4).

**Table 6:** Univariate Cox-regression model hazard ratios and *P* values

		MTV			TLG			bwaMTV			bwaTLG			Interim parameters			
	Clinical stage^a^	glob4	41pc	grad	glob4	41pc	grad	glob4	41pc	grad	glob4	41pc	grad	Deauville score	ΔSUVmax	mqPET	rPET
HR	1.8	2.7	3.1	3.3	2.7	3.1	2.5	2.4	3.4	3.4	2.2	3.2	2.7	5.7	4.1	6.7	9.1
(95% CI)	(0.79–4.1)	(1.1–6.8)	(1.3–7.4)	(1.5–7.2)	(1–1.7)	(1.3–7.8)	(1.2–5.6)	(1.1–5.4)	(1.4–8.4)	(1.6–7.2)	(0.98–5.1)	(1.2–8.4)	(1.3–5.7)	(2.6–12)	(1.9–8.8)	(3.1–14)	(4.2–20)
p	0.163	0.03	0.01	0.003	0.047	0.014	0.019	0.031	0.008	0.002	0.057	0.02	0.01	<0.001	<0.001	<0.001	<0.001

bwa, body weight-adjusted; CI, confidence interval; HR, hazard ratio; grad, method using a gradiant-based lesion growing algorithm; MTV, metabolic tumour volume; mqPET, modified qPET; rPET, ratio PET; SUVmax, maximum standardized uptake value; TLG, total lesion glycolysis; glob4, >SUV4 method; 41pc, 41% isocontour VOI method.

a Early vs. advanced.

**Fig. 5:**
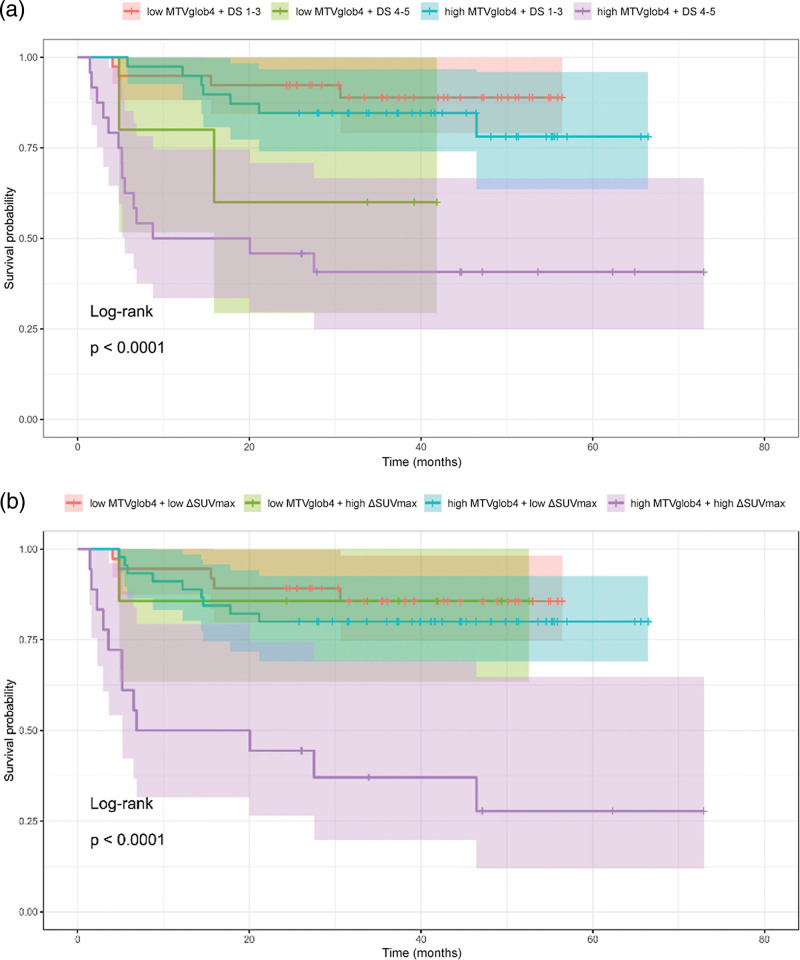
Kaplan–Meier curves of progression-free survival of (a) four subgroups according to low/high MTV and Deauville score 1–3 vs. 4–5 and (b) four subgroups according to low/high MTV and low vs. high ΔSUVmax. MTV, metabolic tumor volume; SUVmax, maximum standardized uptake value.

A combined analysis was performed by forming four groups according to low/high MTV and Deauville scores 1–3 vs. 4–5. Kaplan–Meier curves showed a good survival rate for Deauville scores of 1–3 patients and poor PFS for Deauville scores of 4–5 patients, irrespective of MTV. A similar analysis with ΔSUVmax and MTV resulted in relatively good PFS for all ΔSUVmax negative patients and ΔSUVmax positive patients with low MTV, while ΔSUVmax positive patients with high MTV formed a group with distinctly poor PFS where 11 of 17 patients showed progression within 2 years (Fig. 5).

## Discussion

Several different segmentation algorithms have been used to determine baseline MTV in DLBCL patients. Ilyas *et al*. investigated the SUV ≥ 2.5, the 41%, and the ‘PERCIST’ (≥1.5 × mean SUV + 2 SDs in a 3 cm^3^ right liver lobe VOI) methods [20]. The three segmentation methods yielded different optimal cutoff points for predicting PFS, ranging from 166 to 400 cm^3^ which is similar to our results of 123–345 cm^3^. The same tendency can be observed in MTV measurements of solid tumors as shown by Zhuang *et al*. who performed eight different segmentations in non-small cell lung cancer patients that yielded significantly different MTV values [21]. In a study by Tutino *et al*., MTV measurements of Hodgkin’s lymphoma patients were performed with different thresholds by three different software, there was variability not only between segmentation methods but also between software using the same threshold [22]. The best reproducibility was achieved by the segmentations using fixed thresholds (SUV >2.5 and SUV >4 methods).

Our data indicate that although MTV and TLG yielded only moderately promising prognostic performance and areas under the curve on ROC analyses, the gradient-based segmentation algorithm resulted in the best values, especially in terms of sensitivity and diagnostic accuracy. However, as this latter algorithm is vendor-specific, its widespread use might be limited. TLG did not have better prognostic performance than MTV with the corresponding segmentation methods.

Apart from optimal cutoff points varying in the same patient cohort, MTV also shows a sample dependency as markedly different values can be found among studies performed with the same (or highly similar) segmentation methodology, as in standalone studies referenced in the Ilyas paper and in a meta-analysis by Xie *et al*. and Guo *et al*., with optimal cutoff points ranging between 66 and 601.2 cm^3^ for the SUV ≥ 2.5 methods and between 16.1 and 550 cm^3^ for the 40–41% methods [4,20,23–27].

**Fig. 6:**
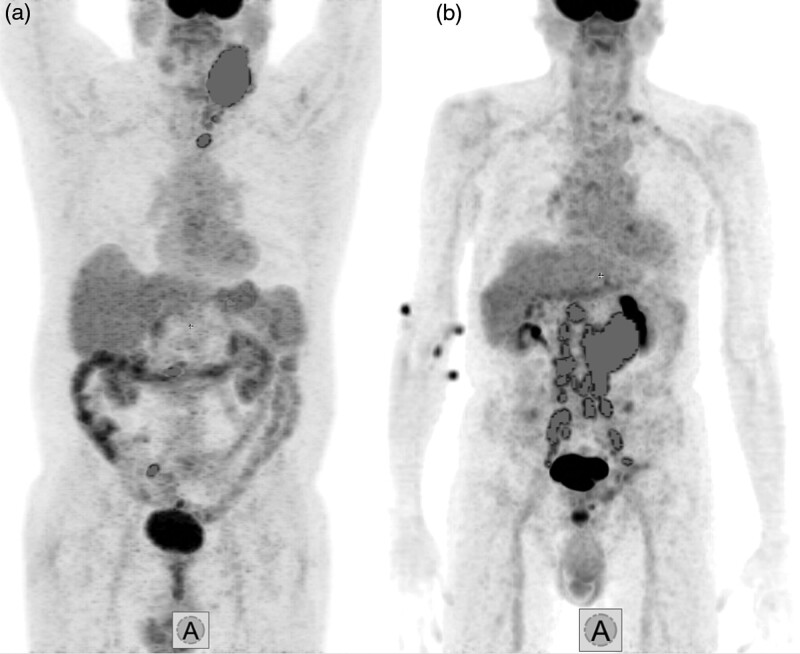
3D MIP PET images with fused MTV VOIs. (a) 92 kg patient with MTV of 189 cm^3^ and bwaMTV of 1.76 who showed no progression during 51 months of follow-up. (b) 54 kg patient with MTV of 292 cm^3^ and bwaMTV of 3.16 who relapsed 7 months after baseline PET (n.b., radiopharmaceutical skin contamination is present in the right cubital area). bwaMTV, body weight-adjusted metabolic tumor volume; MIP, maximum intensity projection; MTV, metabolic tumor volume; VOI, volume of interest.

To the authors’ best knowledge, it is the first time that bwaMTV and TLG values are published. The aim behind the introduction of this normalization was to enable a personalized and more accurate measurement of the impact of tumor burden (normalization to body surface area or lean body mass would also be a feasible option; however, our current dataset did not include patient height in all cases thus making such calculations impossible). Despite bwaMTV and bwaTLG not yielding improved prognostic values over MTV and TLG, respectively, there were a selected few cases where bwaMTV stratified the patient in the correct risk group as opposed to regular MTV (Fig. 6). These values could be further investigated in larger cohorts as their calculation can be easily carried out. Moreover, body surface area could also serve as a parameter for MTV normalization.

ΔSUVmax as a prognostic factor has gained a wider presence in the literature in recent years, with the majority of the studies finding optimal cutoff points around 66% which our finding of 71.22% is close to [12]. Interestingly, in our study, ΔSUVmax evaluation did not result in better prognostic values than the visual Deauville score method in the whole patient cohort.

Semiquantitative ‘Deauville-like’ parameters, especially qPET are gaining more evidence [13–18]. The optimal cutoff for mqPET (using 1 cm^3^ SUVpeak) was 1.32 in our DLBCL cohort which is highly similar to the established qPET (based on a 4-voxel-SUVpeak) cutoff in pediatric Hodgkin’s lymphoma patients and used in the retrospective evaluation of a large German DLBCL study [13,28]. The quotient of the most intensive voxel in the residual lesion – rPET – does not have extensive literature and, to the authors’ best knowledge, this is the first multicentric study to analyze the rPET method in DLBCL patients [16–18]. In our cohort, the optimal cutoff for rPET of 1.54 was higher than the 1.14 and 1.4 values published by Annunziata *et al*. and Toledano *et al*., respectively, and close to Fan and coworkers’ finding of 1.6 [16–18]. In our study, both mqPET and rPET evaluation yielded moderately more accurate prognostic results than Deauville score stratification.

Interim parameters had a higher HR in univariate Cox-regression analyses than baseline volumetric parameters while multivariate Cox-regression analysis resulted in rPET as the only independent predictor of PFS. Also, combined analyses showed that good early treatment response (i.e. Deauville score 1–3) has a higher impact on PFS than baseline MTV. This finding is contradictory to that published by Mikhaeel *et al*. who found that patients with MTV ≥ 400 cm^3^ had a worse prognosis, irrespective of Deauville score on interim scans [29]. Furthermore, in the present study, the combination of baseline MTV and ΔSUVmax enabled us to define a group with a particularly poor prognosis (i.e. patients with high baseline MTV and high ΔSUVmax on interim scan). The discrepancy between our results and those published earlier is most likely due to sample bias, although the multicentric nature of our study might add more reliability to our findings, especially as it had been proven that the international diversity of the original IAEA study had not limited the global applicability of its data [19]. However, the study concluded by Mikhaeel *et al*. contained relatively more patients with stage IV DLBCL (58% vs. 40% in the present report) [29]. Furthermore, a recent review of papers reporting on baseline PET/CT imaging parameters in Hodgkin’s lymphoma and DLBCL found that the majority of published studies investigating baseline MTV are retrospective, heterogenous in methodology, and underpowered [30]. On the other hand, the prognostic impact of visual response assessment using the Deauville five-point scale is more robust and validated [10,12,31]. In that regard, our result of the superior prognostic performance of stratification by Deauville score over MTV is feasible in the context of current scientific pieces of evidence. Furthermore, as pointed out by Barrington *et al*., the standardization of MTV measurement is paramount to gaining a reliable and robust tool in DLBCL risk stratification [32]. Moreover, the ability of patient classification by MTV showed promise in the risk stratification within the low-intermediate and high-intermediate subgroups of NCCN-IPI [33,34]. Furthermore, Baratto *et al*. investigated the change in MTV and TLG between baseline and interim FDG-PET/CT of DLBCL patients and found a prognostic ability of them on PFS and overall survival [35].

In the multicentric setup of our study, the value of visual assessment using the Deauville score is further underlined. Moreover, the prognostic impact of semiquantitative ‘Deauville-like’ parameters (mqPET and rPET) is underlined as well in a multicentric setting. One potential advantage of using SUV ratios with a reference region – that is, mqPET and rPET – over ΔSUVmax could be a partial mitigation of the variability in SUVs of different scanners.

The present study has some limitations. First, the PET/CT devices used at the participating centers had not been cross-calibrated. At present, the reproducibility of SUVs can be supported by the implementation of EARL Harmonization Programme; however, our study had been concluded before its introduction [36]. As radiomics become more prevalent in several imaging research fields, standardization is paramount and the authors would recommend and support collaborations similar to the Image Biomarker Standardization Initiative to make PET imaging parameters more reliable and comparable among centers [37]. Second, as a fixed, empiric cutoff point for MTV values in PFS prediction is yet to be established, our use of ROC-analysis-based optimal thresholds is prone to biases and even with our sample size of 107 patients, it is apparent that cutoff points varied markedly among different MTV segmentation techniques, although neither method showed distinctly superior prognostic performance. Third, information on histopathologic subtypes of DLBCL was not available in the majority of the patients which limits the evaluation of survival data as patients with germinal center B-like DLBCL have significantly better overall survival than those with activated B-like DLBCL [38,39]. Similarly, no analysis of molecular pathology was performed, which would also have added further value to our results in light of recent classifications of DLBCL into molecular subtypes [40–42].

### Conclusion

Baseline MTV values and optimal cutoff points achieved with different segmentation methods varied markedly and showed limited prognostic impact in our multicentric study of DLBCL patients. Interim PET/CT parameters provided more accurate prognostic information with semiquantitative ‘Deauville-like’ parameters (mqPET and rPET) performing best in the present study, as well as more traditional visual response assessment (Deauville score). A combination of baseline MTV and ΔSUVmax allowed the separation of a patient group with a particularly poor prognosis.

## Acknowledgements

We would like to thank all the patients for their participation in the research. We are grateful to the involved medical staff, alongside the International Atomic Energy Agency for their efforts to make this study possible. We thank László Szakács for his contributions to the statistical analysis.

The study was funded and supported by the International Atomic Energy Agency (Coordinated Research Project E1.50.20). No other potential conflict of interest relevant to this article was reported.

### Conflicts of interest

There are no conflicts of interest.
